# High cardiometabolic risk in healthy Chilean adolescents: associations with anthropometric, biological and lifestyle factors

**DOI:** 10.1017/S1368980015001585

**Published:** 2015-05-20

**Authors:** Raquel Burrows, Paulina Correa-Burrows, Marcela Reyes, Estela Blanco, Cecilia Albala, Sheila Gahagan

**Affiliations:** 1 Institute of Nutrition and Food Technology, University of Chile, Avda. El Líbano 5524, Macul 7840390, Santiago de Chile, Chile; 2 Division of Child Development and Community Health, University of California, San Diego, La Jolla, CA, USA

**Keywords:** Metabolic syndrome, Cardiovascular and metabolic risk, Adolescents, Obesity, Sarcopenia, Insulin resistance

## Abstract

**Objective:**

To analyse the prevalence of cardiovascular risk factors in healthy adolescents of low to middle socio-economic status and to study the influence of anthropometric, biological and lifestyle factors on the risk of metabolic syndrome (MetS).

**Design:**

Cross-sectional study. BMI, waist circumference, blood pressure, fat and lean mass (by dual-energy X-ray absorptiometry), TAG, HDL-cholesterol, glucose, insulin, homeostatic model assessment–insulin resistance index (HOMA-IR), food intake and physical activity were measured. Cardiovascular risk factors were defined using the International Diabetes Federation criteria and insulin resistance using HOMA-IR ≥2·6. Bivariate and multivariate regressions examined the associations between MetS and anthropometric, biological and lifestyle factors.

**Setting:**

Observational cohort study including Chilean adolescents, who were part of a follow-up study beginning in infancy.

**Subjects:**

Adolescents aged 16–17 years (*n* 667).

**Results:**

In the sample, 16·2 % had obesity and 9·5 % had MetS. Low HDL-cholesterol (69·9 %), abdominal obesity (33·3 %) and fasting hyperglycaemia (8·7 %) were the most prevalent cardiovascular risk factors. In males, obesity (OR=3·7; 95 % CI 1·2, 10·8), insulin resistance (OR=3·0; 95 % CI 1·1, 8·2), physical inactivity (OR=2·9; 95 % CI 1·1, 7·7) and sarcopenia (OR=21·2; 95 % CI 4·2, 107·5) significantly increased the risk of MetS. In females, insulin resistance (OR=4·9; 95 % CI 1·9, 12·6) and sarcopenia (OR=3·6; 95 % CI 1·1, 11·9) were significantly associated with MetS.

**Conclusions:**

High prevalences of obesity, abdominal obesity, dyslipidaemia, fasting hyperglycaemia and MetS were found in healthy adolescents. In both sexes, sarcopenia and insulin resistance were important risk factors of MetS. Promotion of active lifestyles at the school level and regulation of the sale of energy-dense foods are needed.

The growing prevalence of overweight and obesity has led to higher rates of type 2 diabetes and CVD, both of which may be preceded by the metabolic syndrome (MetS) during childhood and adolescence^(^ ^1^ ^,^ ^2^ ^)^. The MetS is a clustering of cardiometabolic disorders, including abdominal obesity, fasting hyperglycaemia, dyslipidaemia and hypertension, and it allows early identification of individuals with future risk of type 2 diabetes and cardiovascular disorders^(^ ^3^ ^,^ ^4^ ^)^. Cardiovascular and metabolic diseases currently account for the greatest loss of healthy life years, not to mention the economic burden, which is expected to keep growing due to higher life expectancy rates^(^ ^5^ ^,^ ^6^ ^)^. In children and adolescents, MetS is associated with inflammation, insulin resistance (IR), obesity, and a Western diet and sedentary lifestyle^(^ ^7^ ^–^ ^10^ ^)^. IR is the most common metabolic alteration related to obesity and, similarly, is an important link between obesity and MetS, type 2 diabetes and cardiovascular complications^(^ ^7^ ^,^ ^9^ ^)^. Moreover, during childhood and adolescence, a negative association between muscular fitness and cardiometabolic risk has been found^(^ ^11^ ^)^.

The Organization for Economic Co-operation and Development and the WHO have recognized unhealthy diets and physical inactivity as strong early determinants of obesity and non-communicable chronic diseases in adulthood^(^ ^12^ ^)^. In the last three decades, Chile underwent a remarkable nutritional transition, with increasing Western dietary patterns and inactive lifestyles resulting in high prevalences of obesity, type 2 diabetes and CVD, particularly in low socio-economic levels^(^ ^13^ ^–^ ^15^ ^)^. From 1986 to 1998, the odds of obesity increased sixfold in boys and fourfold in girls aged 6–16 years^(^ ^16^ ^)^. Likewise, high rates of IR (53 %) and MetS (30 %) have been documented among obese Chilean children and adolescents aged 4–16 years^(^ ^17^ ^,^ ^18^ ^)^. A more recent study in children and adolescents attending public schools showed prevalence rates of obesity, MetS and IR of 16·1 %, 7·3 % and 25·9 %, respectively^(^ ^19^ ^)^. Furthermore, in the 2011 Physical Activity System for the Assessment of Educational Quality (SIMCE_EF), a national standardized test administered to all 8th grade students, an obesity prevalence of 16 % was confirmed, whereas 93 % and 78 % of assessed students had impaired muscle functioning and low aerobic capacity, respectively^(^ ^20^ ^)^.

The aim of the present study was to analyse the prevalence of cardiovascular risk factors and the influence of anthropometric, biological and lifestyle factors on odds for MetS in a cohort of healthy Chilean adolescents, who were part of randomized controlled trial of Fe to prevent Fe deficiency in infancy and a longitudinal follow-up study.

## Methods

### Study design and population

We studied 667 adolescents aged 16–17 years of low to middle socio-economic status (SES), living in urban Santiago, who were part of a follow-up study beginning in infancy. The infants, recruited at 4 months, were healthy, full-term singleton infants weighing 3 kg or more at birth. At 6 months, those who were not Fe deficient were randomized to receive Fe supplementation or usual nutrition from 6 to 12 months. They were assessed for developmental outcomes in infancy and at 5, 10 and 16 years. At 16 years, they were also assessed for obesity risk and the presence of cardiovascular risk factors^(^ ^21^ ^)^. The study was approved by the institutional review boards of the University of Michigan, the Institute of Nutrition and Food Technology (University of Chile), and the University of California, San Diego. Participants and their primary caregiver provided informed and written consent, which was obtained according to the norms for Human Experimentation, Code of Ethics of the World Medical Association (Declaration of Helsinki, 1995).

### Measurements

#### Anthropometry and body composition

A research physician used standardized procedures to measure the adolescent’s height to the nearest 0·1 cm using a Holtain stadiometer and weight to the nearest 0·1 kg using a Seca scale. BMI (kg/m^2^) was calculated and obesity status was calculated according to WHO references. Waist circumference (WC) was measured with non-elastic flexible tape at the high point of the iliac crest around the abdomen and recorded to 0·1 cm. Measurements were taken twice, with a third measurement if the difference between the first two exceeded 0·3 kg for weight, 0·5 cm for height and 1·0 cm for waist. Dual-energy X-ray absorptiometry was used to measure fat mass (%) and fat-free mass (%). Fat-free mass index was estimated according to Wells and Fewtrell^(^ ^22^ ^)^. Fat-free mass index values were expressed as a percentage; values ≤25th percentile in our sample, adjusted for sex, were defined as relative sarcopenia. In the remainder of the paper we use the term ‘sarcopenia’ to refer to relative sarcopenia.

#### Additional cardiovascular risk markers

After 15 min at rest and before the other physical evaluations, systolic and diastolic blood pressures (SBP and DBP) were measured three times on the non-dominant arm using a standard mercury sphygmomanometer; the average value was used for analyses. Fasting serum total glucose, cholesterol, TAG, HDL-cholesterol (HDL-C), insulin, adiponectin and high-sensitivity C-reactive protein (hs-CRP) levels were performed after a 12 h overnight fast. RIA (Diagnostic Products Corporation, Los Angeles, CA, USA) was used for insulin and adiponectin determinations. hs-CRP was measured with a sensitive latex-based immunoassay. Glucose was measured with an enzymatic colorimetric test (QCA S.A., Amposta, Spain) and cholesterol profile (HDL-C and TAG, mg/dl) were determined by dry analytical methodology (Vitros^®^; Ortho Clinical Diagnostics Inc., Raritan, NJ, USA). Homeostatic model assessment–insulin resistance index (HOMA-IR) was calculated and a value of 2·6 was considered to indicate IR^(^ ^23^ ^)^. Values of hs-CRP ≥1·1 mg/l (75th percentile in our sample) were considered low-grade systemic (LGS) inflammation and values of adiponectin ≤7·9 µg/ml (25th percentile in our sample) were considered to be low.

### Definition of metabolic syndrome and cardiovascular risk factors

In order to compare our results with findings from other national and international studies, MetS was diagnosed based on the 2007 International Diabetes Federation (IDF) consensus statement on the clinical definition of the MetS in the paediatric age range. These criteria include having central obesity plus two of the other four factors using the following definitions: (i) abdominal obesity (WC≥80 and ≥90 cm in females and males, respectively); (ii) high blood pressure (SBP≥130 mmHg, DBP≥85 mmHg); (iii) hypertriacylglycerolaemia (TAG≥150 mg/dl); (iv) low HDL-C (≤50 and ≤40 mg/dl in female and male adolescents, respectively); and (v) fasting hyperglycaemia (total glucose≥100 mg/dl)^(^ ^24^ ^)^. The cardiovascular risk factors included each criterion of the IDF definition of MetS. The new MetS definition by the IDF and American Heart Association/National Heart, Lung, and Blood Institute (AHA/NHLBI)^(^ ^25^ ^)^ was used to assess potential differences in the prevalence of MetS.

### Food intake and physical activity

Food intake and physical activity habits were measured using validated and standardized self-report questionnaires and scored from 0 to 10, with higher scores denoting better quality of food intake or more physical activity^(^ ^26^ ^,^ ^27^ ^)^. The questionnaires were administered by a researcher during the half-day assessment. The quality of food intake was measured by the amounts of saturated fat, fibre, sugars and salt in the food items. We applied cut-offs from a national reference standard^(^ ^26^ ^)^ to classify the eating habits of participants into three groups: unhealthy (score≤4·30), intermediate (score=4·31–5·99) and healthy (score≥6·00). Physical activity was measured by the total amount of time devoted to sedentary activities, recreational games, active commuting, and weekly scheduled exercise either school or non-school organized. Participants with a score ≤3·00 were classified as physically inactive, those with a score ≥6·00 were physically active, and those in between were moderately active^(^ ^26^ ^)^.

### Statistical analysis

Statistical analysis included Student’s *t* test and Wilcoxon’s rank-sum test for comparison of mean or median values of anthropometric, cardiometabolic and lifestyle variables. The *χ* ^2^ test was used for comparison of categorical variables. Given biological and lifestyle differences between male and female adolescents, we tested interactions between sex, anthropometric and biological factors on development of MetS by using two-way ANOVA. The interaction with sex was significant at *α* level of 0·05 (data not shown) and, therefore, we stratified the analysis. After performing unadjusted logistic regressions to test the associations between MetS and biological (LGS inflammation, low adiponectin and IR), anthropometric (obesity and relative sarcopenia) and lifestyle (physical inactivity and unhealthy food intake) variables, we used multiple logistic regressions to assess the relationship between the risk of MetS and the variables significantly associated with MetS. Three models were estimated. The first one included biological variables. In the second model, anthropometric variables were added. Finally, a fully adjusted model contained all mentioned covariates with the addition of lifestyle factors. Data were analysed using the statistical software package Stata for Windows version 12·0. A *P* value of <0·05 denoted statistical significance.

## Results

The participants were 16·8 (sd 0·3) years old, and 52·2 % male. The prevalence of obesity was 16·2 % (95 % CI 13·4, 18·9 %). At least one cardiovascular risk factor was present in 79 % of the participants and 9·5 % (95 % CI 7·2, 11·7 %) met criteria for the MetS. When the MetS was diagnosed by using the new IDF and AHA/NHBLI definition, the prevalence increased to 9·8 % (95 % CI 7·5, 12·0 %).

Table 1 shows the anthropometric, cardiometabolic and lifestyle characteristics of adolescents in the sample by sex. Males had significantly higher mean values of blood pressure (SBP and DBP), glycaemia and physical activity score, and lower levels of adiponectin and HDL-C, compared with females. Prevalence of low adiponectin was significantly higher in males, while physical inactivity was significantly higher in females (all *P*<0·001).

**Table 1:** Anthropometric, cardiometabolic and lifestyle profile in healthy male and female adolescents aged 16–17 years from Santiago, Chile (*n* 667)

	Males (*n* 348)		Females (*n* 319)		Overall sample (*n* 667)	
	Mean or %	sd or 95 % CI	Mean or %	sd or 95 % CI	Mean or %	sd or 95 % CI
Continuous variables (mean and sd)						
Age (years)	16·8	0·2	16·8*	0·3	16·8	0·3
BMI (*Z*-score)	0·58	1·2	0·73*	1·2	0·65	1·2
WC (cm)	81·3	11·1	81·2	11·8	81·2	11·4
Total fat mass (%)	22·4	8·8	36·3***	7·5	29·0	10·5
Total fat-free mass (%)	74·5	9·0	59·9***	7·5	67·6	10·8
SBP (mmHg)	115·4	10·4	108·8***	9·7	112·2	10·6
DBP (mmHg)	70·8	7·0	67·6***	6·7	69·3	7·0
Glucose (mg/dl)	90·4	9·6	86·5***	9·0	88·5	9·5
Insulin (µUI/ml)	7·8	5·7	8·4	5·3	8·1	5·5
HOMA-IR	1·8	1·4	1·8	1·2	1·8	1·3
TAG (mg/dl)	88·4	52·4	88·2	47·6	88·3	50·2
HDL-C (mg/dl)	38·0	10·1	42·5***	10·7	40·1	10·6
hs-CRP (mg/l)	1·0	2·2	1·0	1·8	1·0	1·9
Adiponectin (µg/ml)	10·7	5·2	13·0***	5·6	11·8	5·5
Physical activity (score)†	5·0	2·0	3·0***	1·0	4·0	2·0
Food intake (score)	5·2	1·2	5·3	1·3	5·2	1·2
Categorical variables (% and 95 % CI)						
LGS inflammation	20·1	15·8, 24·3	24·1	19·4, 29·9	22·0	18·9, 25·2
Low adiponectin	24·1	19·6, 28·6	12·2***	8·6, 15·8	18·4	15·5, 21·4
Insulin resistance	16·0	12·1, 19·9	16·6	12·5, 20·7	16·5	13·6, 19·3
Obesity	15·8	11·9, 19·6	17·0	12·8, 21·2	16·1	13·4, 19·0
Sarcopenia	24·9	20·4, 29·5	25·4	20·6, 30·2	25·1	21·9, 28·4
Physical inactivity	24·4	19·8, 29·0	57·9***	52·4, 63·3	40·8	37·0, 44·6
Unhealthy food intake	23·2	18·8, 27·7	24·5	19·7, 29·2	23·8	20·6, 27·0

WC, waist circumference; SBP, systolic blood pressure; DBP, diastolic blood pressure; HOMA-IR; homeostatic model assessment–insulin resistance index; HDL-C, HDL-cholesterol; hs-CRP, high-sensitivity C-reactive protein; LGS, low-grade systemic. LGS inflammation: hs-CRP ≥75th percentile (hs-CRP≥1·1 mg/l). Low adiponectin: adiponectin ≤25th percentile (adiponectin ≤7·9 µg/ml). Insulin resistance: HOMA-IR ≥2·6. Obesity: BMI *Z*-score ≥2. Relative sarcopenia: % fat-free mass index ≤25th percentile. Physical inactivity: physical activity score ≤3. Unhealthy food intake: food intake score ≤3·3. Significant difference between males and females (Student’s *t* test, Wilcoxon rank-sum test or *χ* ^2^ test): *P<0·05, ***P<0·001.

† Expressed as median and interquartile range.

**Fig. 1:**
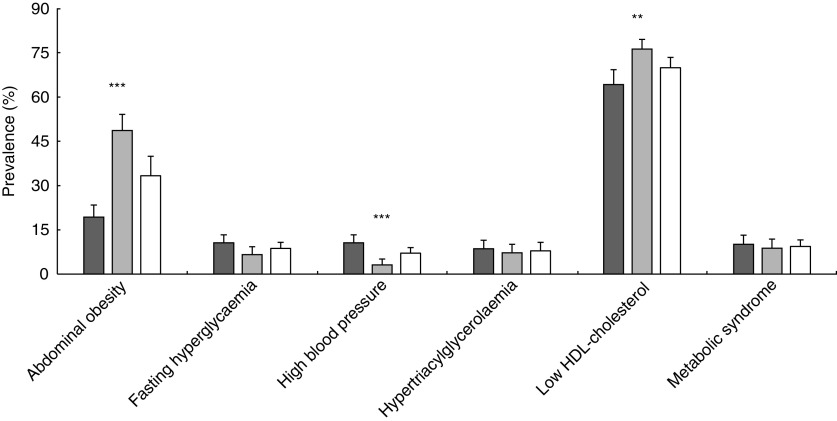
Prevalence rates of cardiovascular risk factors and metabolic syndrome (with 95 % confidence intervals represented by vertical bars) among adolescents (*n* 667) aged 16–17 years (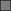, males; 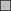, females; 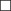, overall sample) from Santiago, Chile. Significant difference between males and females (Pearson’s *χ* ^2^ test): **P<0·01, ***P<0·001

In the overall sample, low HDL-C (69·9 %; 95 % CI 66·4, 73·4 %) and abdominal obesity (33·3 %; 95 % CI 29·7, 36·9 %) were the most prevalent cardiovascular risk factors (Fig. 1). Fasting hyperglycaemia prevalence was 8·7 % (95 % CI 6·5, 10·8 %). After controlling sex, males had a higher prevalence of elevated blood pressure than females (*P*<0·001). Females had a higher prevalence of abdominal obesity (*P*<0·001) and low HDL-C (*P*<0·01) than males.

Tables 2 and 3 present the estimated associations between biological, anthropometric and lifestyle factors with MetS in males and females, respectively. Unadjusted and adjusted odds ratios along with 95 % confidence intervals are provided. In males (Table 2), we found a statistically significant association between MetS and the following variables: LGS inflammation, low adiponectin, obesity, IR, sarcopenia and physical inactivity. After full adjustments, only associations with the four last covariates remained significant. In this model, the major risk factor was sarcopenia (OR=21·2; 95 % CI 4·18, 107·5), followed by obesity (OR=3·7; 95 % CI 1·23, 10·8). Among females (Table 3), MetS was significantly related with low adiponectin, obesity, IR, sarcopenia and physical inactivity; however, only IR (OR=4·96; 95 % CI 1·95, 12·6) and sarcopenia (OR=3·61; 95 % CI 1·10, 11·9) remained significantly associated with higher odds of MetS in the model with full adjustments.

**Table 2:** Influence of biological, anthropometric and lifestyle factors (dependent variables) on the risk of metabolic syndrome (independent variable) in healthy male adolescents aged 16–17 years from Santiago, Chile (*n* 348)

			Adjusted OR					
	Unadjusted OR		Model 1		Model 2		Model 3	
Variable	OR	95 % CI	OR	95 % CI	OR	95 % CI	OR	95 % CI
LGS inflammation	2·65**	1·26, 5·58	3·14**	1·34, 7·31	1·73	0·63, 4·78	1·65	0·58, 4·78
Low adiponectin	2·65**	1·28, 5·45	1·69	0·75, 3·79	1·53	0·56, 4·15	1·59	0·57, 4·48
Insulin resistance	10·26***	4·83, 21·8	10·37***	4·66, 23·1	3·20*	1·20, 8·54	3·00*	1·10, 8·23
Obesity	28·29***	12·1, 66·1	–	–	3·42*	1·23, 9·51	3·74*	1·23, 10·8
Relative sarcopenia	79·13***	18·4, 339·8	–	–	25·9***	5·22, 128·9	21·2***	4·18, 107·5
Physical inactivity	4·91***	2·38, 10·1	–	–	–	–	2·94*	1·12, 7·72
Unhealthy food intake	0·82	0·34, 1·96	–	–	–	–	–	–
Likelihood ratio (*χ* ^2^)			44·95***		107·1***		112·0***	
Hosmer–Lemeshow			6·20 (*P*=0·19)		5·34 (*P*=0·14)		5·33 (*P*=0·50)	
Correctly classified (%)			89·7		91·1		92·5	

LGS, low-grade systemic; –; non-observed; hs-CRP, high-sensitivity C-reactive protein; HOMA-IR; homeostatic model assessment–insulin resistance index. Model 1, biological variables; model 2, model 1 plus anthropometric variables; model 3, model 2 plus lifestyle factors. LGS inflammation: hs-CRP ≥75th percentile (hs-CRP≥1·1 mg/l). Low adiponectin: adiponectin ≤25th percentile (adiponectin≤7·9 µg/ml). Insulin resistance: HOMA-IR ≥2·6. Obesity: BMI *Z*-score ≥2. Relative sarcopenia: % fat-free mass index ≤25th percentile. Physical inactivity: physical activity score ≤3. Unhealthy food intake: food index score ≤3·3. Statistical significance: *P<0·05, **P<0·01, ***P<0·001.

**Table 3:** Influence of biological, anthropometric and lifestyle factors (dependent variables) on the risk of metabolic syndrome (independent variable) in healthy female adolescents aged 16–17 years from Santiago, Chile (*n* 348)

			Adjusted OR					
	Unadjusted OR		Model 1		Model 2		Model 3	
Variable	OR	95 % CI	OR	95 % CI	OR	95 % CI	OR	95 % CI
LGS inflammation	1·55	0·67, 3·59	–	–	–	–	–	–
Low adiponectin	3·35**	1·36, 8·25	2·46	0·92, 6·58	1·86	0·63, 5·50	1·78	0·59, 5·39
Insulin resistance	9·15***	4·01, 20·9	8·31***	3·60, 19·2	4·90***	1·94, 12·4	4·96**	1·95, 12·6
Obesity	11·0***	4·75, 25·0	–	–	2·85	0·86, 9·43	2·64	0·81, 8·62
Relative sarcopenia	9·40***	3·96, 22·4	–	–	3·50*	1·05, 11·7	3·61*	1·10, 11·9
Physical inactivity	2·92*	1·15, 7·41	–	–	–	–	2·44	0·87, 6·88
Unhealthy food intake	1·04	0·42, 2·53	–	–	–	–	–	–
Likelihood ratio (*χ* ^2^)			29·96***		50·7***		53·8***	
Hosmer–Lemeshow			6·20 (*P*=0·19)		2·51 (*P*=0·64)		5·76 (*P*=0·33)	
Correctly classified (%)			91·2		92·2		92·2	

LGS, low-grade systemic; –, non-observed; hs-CRP, high-sensitivity C-reactive protein; HOMA-IR; homeostatic model assessment–insulin resistance index. Model 1, biological variables; model 2, model 1 plus anthropometric variables; model 3, model 2 plus lifestyle factors. LGS inflammation: hs-CRP ≥75th percentile (hs-CRP≥1·1 mg/l). Low adiponectin: adiponectin ≤25th percentile (adiponectin≤7·9 µg/ml). Insulin resistance: HOMA-IR ≥2·6. Obesity: BMI *Z*-score ≥2. Relative sarcopenia: % fat-free mass index ≤25th percentile. Physical inactivity: physical activity score ≤3. Unhealthy food intake: food index score ≤3·3. Statistical significance: *P<0·05, **P<0·01, ***P<0·001.

## Discussion

The present study analysed the influence of anthropometric, biological and lifestyle factors on cardiovascular and metabolic risk, as measured by the MetS, in a sample of healthy Chilean adolescents of middle to low SES. We found a high prevalence of obesity (16·2 %) and MetS (9·4 %); differences by sex were non-significant. The prevalence of obesity was higher than the figures reported by the 2013 Global School-Based Student Health Survey (9·9 %), as well as the 2014 Chilean National Food Consumption Survey (13·7 %), for the age-matched general population^(^ ^28^ ^,^ ^29^ ^)^, but it is very similar to obesity rates in adolescents living in vulnerable socio-economic conditions^(^ ^30^ ^)^. Likewise, results from two cross-sectional studies conducted in Chile in 10- to 15-years-olds and 22- to 28-year-olds from low to middle SES were also very similar to our results, including the prevalence of MetS (7·3 % and 10 %, respectively)^(^ ^19^ ^,^ ^30^ ^)^.

It is worth noting that the prevalence of MetS in Chilean adolescents is remarkably higher than that found in a cohort of 16-year-olds from Finland (1·7 %) and 16–19-year-olds from South Korea (3·1 %) using the same diagnosis criteria^(^ ^31^ ^,^ ^32^ ^)^. Finnish adolescents showed lower mean values of BMI, WC and HOMA-IR than their Chilean peers. Similarly, South Koreans showed a lower prevalence of obesity (8·4 %), abdominal obesity (11·2 %), low HDL-C (33·2 %) and fasting hyperglycaemia (4·8 %) than Chilean adolescents. Differences in the level of human development may in part explain differences in the prevalence of cardiovascular risk factors and MetS. Finland and South Korea are both countries with a very high level of human development. Even though Chile recently joined this category, when adjustments are made to account for inequality, which is the actual level of human development, we see that differences at the education, health and lifestyle levels are particularly pronounced^(^ ^15^ ^,^ ^33^ ^)^.

Although inflammation in males and low adiponectin levels in both sexes were significantly associated with meeting criteria for the MetS, these associations were lost after accounting for other influences. Similar to findings from other studies, their influence over the risk of MetS might be mediated by IR^(^ ^34^ ^,^ ^35^ ^)^. In both sexes, IR was significantly and independently associated with higher odds of MetS. Thus, our results confirm the previously reported association of IR with early cardiovascular risk^(^ ^8^ ^,^ ^9^ ^,^ ^19^ ^)^. In a previous study in a large clinical sample of Chilean overweight and obese adolescents, we found that HOMA-IR ≥3·3 was significantly associated with MetS^(^ ^23^ ^)^. Similarly, in Mexican obese children and adolescents, an increased degree of IR was associated with a higher prevalence of each of the components of MetS and a higher risk of MetS, regardless of age and sex^(^ ^36^ ^)^. In males from our sample, obesity was independently associated with higher risk of MetS. In females, however, obesity lost its influence after accounting for IR. This could be due to an underestimation of total body fat and metabolic risk when obesity is diagnosed by BMI, or the influence might be mediated by IR^(^ ^37^ ^,^ ^38^ ^)^.

Relative sarcopenia showed a strong, direct and independent association with the risk of MetS in both sexes, which is consistent with results found in other contexts. In children and adolescents, higher levels of muscular fitness have been inversely associated with obesity, IR, clustered cardiometabolic risk and inflammation^(^ ^39^ ^–^ ^41^ ^)^. Likewise, muscular and cardiorespiratory fitness were independently associated with metabolic risk in European adolescents, according to the HELENA (Healthy Lifestyle in Europe by Nutrition in Adolescence) study^(^ ^42^ ^)^. It is well known that physical inactivity increases the risk of several adverse health conditions, including major non-communicable diseases such as CHD, type 2 diabetes, and breast and colon cancers, not to mention reductions of life expectancy^(^ ^43^ ^)^. However, in our sample, physical inactivity was independently associated with MetS only in males. Several studies suggest that because males and females have different levels of physical activity, and the variables associated with activity levels are not consistent across the sexes, there could be differences in the impact of physical inactivity on metabolic risk^(^ ^44^ ^,^ ^45^ ^)^. In the present study, physical activity was assessed by self-report of the time allocated to repetitive, planned physical activity during the week, regardless of the intensity or type of exercise. This may have led to underestimation of the impact of physical inactivity on metabolic risk in females.

### Limitations and strengths

The current research has some weaknesses that should be considered. Our sample is not representative of the Chilean adolescent population, as it was made up of adolescents from low to middle SES living in urban Santiago. Participants were drawn from a longitudinal cohort that began in infancy as a preventive trial of Fe-deficiency anaemia. The inclusion criteria mandated that all participants weigh ≥3 kg and be born full-term to healthy mothers. Thus, our results cannot be generalized to a larger population. However, our findings may be equally relevant for a number of reasons. According to the Chilean National Health Survey, the prevalence of obesity, type 2 diabetes and CVD is significantly higher among individuals from low to middle SES^(^ ^15^ ^)^. On the other hand, low- to middle-SES Chilean adolescents are highly exposed to risk factors that have a direct effect on the development of non-communicable diseases, including physical inactivity and processed foods full of fat and sugar^(^ ^26^ ^,^ ^46^ ^,^ ^47^ ^)^. A further limitation is the cross-sectional nature of the study, which limits the ability to draw conclusions related to the temporality of these relationships. Future studies should aim to longitudinally explore links between biological and environmental factors and MetS, which may become clearer over time. Finally, physical activity was not assessed with an objective measurement^(^ ^48^ ^)^. However, we used a questionnaire that was validated using accelerometry-based activity monitors in a sample of adolescents from urban Santiago and from all SES groups^(^ ^27^ ^)^. Muscle mass was assessed using dual-energy X-ray absorptiometry rather than whole-body MRI^(^ ^49^ ^)^. Nevertheless, dual-energy X-ray absorptiometry methodology and fat-free mass index have better sensitivity than BMI for estimating the ratio between muscle and fat tissue^(^ ^22^ ^,^ ^50^ ^)^.

In spite of these limitations, the research provides results that confirm an increased cardiovascular and metabolic risk in a sample of adolescents who grew up during a rapid nutritional and epidemiological transition. These results may be useful for middle-income countries and populations going through similarly rapid nutritional and epidemiological transitions, including several countries in Latin America, Eastern Europe and South-East Asia, and immigrants and ethnic minorities in the USA and Western Europe^(^ ^51^ ^,^ ^52^ ^)^. A further important contribution is the confirmation that reduced muscle development might be considered a relevant risk factor preceding non-communicable diseases and as important as obesity and IR. A major public health challenge will be to increase the number of years free of disability in a generation exposed to such dramatic changes in the food and physical activity environment^(^ ^53^ ^)^. Public policies and programmes at the school level that promote active lifestyles in children and adolescents, and regulate the sale of energy-dense foods, are sorely needed. Promotion of patterns lifestyle that include higher dairy consumption along with more adequate breakfast, high-fibre foods, more frequent meals and higher physical activity among children, adolescents and their families should be considered as one of the components of any obesity prevention initiative^(^ ^54^ ^)^.
